# PW01-033 – Phenotype – genotype in Armenian children with FMF

**DOI:** 10.1186/1546-0096-11-S1-A86

**Published:** 2013-11-08

**Authors:** G Amaryan, T Sarkissian, T Sarkissian, H Hayrapetyan, H Hayrapetyan, A Tadevosyan, A Tadevosyan

**Affiliations:** 1 National Pediatric Centre for Familial Mediterranean Fever of "Arabkir" Joint Medical Centre - Institute of Child and Adolescent Health, Yerevan State Medical University, Armenia; 2 Centre of Medical Genetics and Primary Health Care, Yerevan, Armenia; 3 Yerevan State Medical University, Yerevan, Armenia

## Introduction

Familial Mediterranean Fever (FMF) is an ethnic disease for Armenian population and represents a significant health care problem. Frequency of carriers of *MEFV* mutations is 1:3, and the prevalence of FMF is rather high (14-100:10000). During the period between 2003 and 2012 there was a 4.5-fold increase of the total number of children with FMF. Many of these cases have severe clinical picture and atypical course, which may complicate timely diagnosis.

## Objectives

To establish phenotype-genotype correlations in Armenian children with FMF

## Methods

We analyzed a group of 715 children with FMF (438 boys and 277 girls, mean age: 8.64±0.17), including sporadic (51.8%) and familial cases (48.2%). The diagnosis was based on the Tel-Hashomer criteria, the “Guidelines for the genetic diagnosis of hereditary recurrent fevers”(2011) and molecular-genetic detection of 12 *MEFV* mutations common for Armenians. The statistical analysis was performed using Epi-Info 2000 software. For comparison of two nominal variables in table “two by two” Yaet’s corrected for continuity chi-square test was used, significance level p<0.05.

## Results

FMF manifestation during the first decade of life were determined in 95.2% of patients (mean age of onset 3.5±0.1, mean age of diagnosis 5.25±0.15). FMF onset before 5 years was in 78.6%. In 96.5% of patients severe phenotype with polyserositis was associated with *MEFV* mutations in exon 10 (M694V- 58.1%; V726A- 20.4%; M680I- 15.7%). In contrast, mild or atypical FMF phenotypes without polyserositis were found in heterozygous carriers of one *MEFV* mutation in exons 2 and 3 (E148Q and P369S, respectively). We suggest that our patients with FMF developed pleurisy more frequently (81.7%), also myalgia (37.5%), pericarditis (13.8%), and rare skin lesions (13.4%) mostly as erysipelas-like erythema, ELE, (10.8%) in comparison with other populations (Jews, Turks, Arabs).

Among concurrent pathologies, we found Henoch-Shonlein purpura (HSP,1.5%), protracted febrile myalgia (PFM, 2.7%) as well as the higher than expected frequencies for juvenile idiopathic arthritis (JIA, 4.7%), non-amyloid renal involvements (NARI, 1.1%) and ulcerative colitis/Crohn’s disease (UC/CD, 1%). The development of serositis, splenomegaly, ELE and vasculitis was associated with *M694* homozygous and compound-heterozygous genotypes. Early manifestation and severe FMF attacks were detected in patients carrying *M694V* genotypes, whereas the M680I and V726A mutations were associated with relatively mild clinical features. Adhesive intestinal obstruction (AIO), was detected in 3.2% of patients, in some cases as the first and only FMF manifestation, especially in the *M694V* carriers.

## Conclusion

Taking into account the high prevalence of FMF in Armenia, *MEFV* mutation screening is recommended not only for patients with atypical symptoms resembling FMF, but also for patients with FMF- associated vasculitis (HSP, PFM) as well as co-existed immune diseases (JIA, NARI, UC/CD) and AIO. In addition to improving the early diagnosis of FMF and preventing the development of amyloidosis, this can be especially important in the patients resistant to conventional treatments for the aforementioned FMF-associated and other concurrent diseases.

## Disclosure of interest

None declared

